# Validating Digital Scribes: A Scoping Review of Evaluation Practices and Clinical Use

**DOI:** 10.1007/s10916-026-02392-3

**Published:** 2026-04-24

**Authors:** Ekin Kerimoğlu, Fleur V. Notermans, Milou E. W. M. Silkens, Marleen de Mul, Kees C. T. B. Ahaus, Robert M. A. van der Boon

**Affiliations:** 1 Erasmus School of Health Policy and Management (ESHPM), Erasmus University Rotterdam, Bayle Building, Burgemeester Oudlaan 50, Rotterdam, 3062PA The Netherlands; 2 Dijkzigt, Erasmus Medical Center (Erasmus MC), Dr. Molewaterplein 40, Rotterdam, 3015GD The Netherlands; 3 Department of Cardiology, Cardiovascular Institute, Erasmus Medical Center (Erasmus MC), Dr. Molewaterplein 40, Rotterdam, 3015GD The Netherlands

**Keywords:** Digital scribes, Clinical documentation, Validation frameworks, Artificial intelligence in healthcare, Large language models (LLMs), Ambient listening

## Abstract

Clinical documentation accounts for a substantial share of clinicians’ working time, contributing to administrative burden and reduced patient-facing care. Artificial intelligence has stimulated the development of digital scribes that combine speech recognition (ASR) and large language models (LLMs) to generate clinical notes from patient–provider conversations with the aim to automate and support this process and reduce this burden. This scoping review explores how digital scribes are currently validated, both technical and clinical, and whether they reliably support clinical workflows. Using the Technology Readiness Level (TRL) framework, we show that most systems remain in early development stages (typically TRL 3&4), with only a small number progressing to workflow integration. While digital scribes show potential to improve documentation efficiency, validation methods are highly heterogeneous, most studies rely on simulated or retrospective data, and real-world testing is limited. Consequently, cross-system comparisons and conclusions about clinical performance remain limited. We identified three motivational frames: human-, performance-, and system-oriented, which shape evaluation practices and outcome expectations. These findings suggest that successful implementation depends not only on scribes’ technical capability but also on alignment with clinical needs and documentation styles. Overall, our review underscores the need for standardised validation frameworks and prospective real-world studies to ensure that digital scribes progress beyond their current low TRL and move from experimental promise to safe, effective, and sustainable integration into clinical care.

**Supplementary Information:**

The online version contains supplementary material available at 10.1007/s10916-026-02392-3.

## Introduction

Since the widespread adoption of electronic health records (EHRs), clinicians spend up to half their working hours on clinical documentation [1]. This burden is linked to burnout, decreased job satisfaction, and documentation errors [2, 3]. Medical scribes have long served as a way to reduce the burden by documenting consultations, updating EHRs, and assisting with administrative tasks [4]. However, recent advances in generative artificial intelligence (AI), particularly large language models (LLMs), offer a new automated alternative: the digital scribe.

Digital scribes combine automatic speech recognition (ASR) and LLMs to capture patient-provider conversations and generate clinical documentation [2]. ASR refers to continuously recording audio, detecting relevant speech and filtering background noise [5]. Despite its technical maturity, ASR alone has seen limited clinical uptake due to narrow utility and reliance on manual post-processing [6]. LLMs overcome these limitations by performing advanced language tasks such as summarization [7], enabling digital scribes to transcribe and produce coherent, context-aware notes. This could reduce documentation burden and support more natural, screen-free interactions. A relevant consideration, given evidence that screen use can hinder communication with patients and decrease relational quality [3, 5].

Validating digital scribes is essential to ensure they reliably support clinical workflows. However, validation remains limited, methods vary considerably, real-world studies are scarce, and existing frameworks for assessing usability, safety, and efficacy are often lacking or only partially applied [1]. The choice of validation strategy directly shapes the quality and credibility of results, which in turn influences clinical acceptance and integration. While LLM-based systems enhance transcription accuracy, their ability to interpret and summarize interactions may lead to concerns about bias, misrepresentation, and unintended changes in clinical communication [8]. Digital scribes also introduce patient-facing considerations raising questions about access, review, and correction of the generated content. Thus, integration into healthcare settings requires thoughtful, context-specific implementation and careful evaluation to ensure digital scribes support rather than undermine patient-provider interactions.

This scoping review examines how digital scribes combining ASR and LLMs for patient-provider interactions are being developed, validated, and integrated into clinical workflows to support real-time documentation and reduce administrative burden. It synthesizes the current evidence on validation approaches and both technical and clinical performance of these systems, with the aim of assessing their current utility and reliability in healthcare settings. By providing this overview, this review highlights opportunities and challenges in current practice and outlines considerations needed to ensure safe, effective, and sustainable adoption.

## Methods

A scoping review was performed following the Preferred Reporting Items for Systematic Reviews and Meta-Analysis guidelines, using the extension for scoping reviews (PRISMA-ScR) [9]. A scoping review was chosen for this study to identify available evidence in this domain, summarize existing research, highlight gaps in the literature, and propose directions for future inquiry. As PROSPERO does not accept registrations for scoping reviews, no protocol was registered.

### Search Strategy and Selection Criteria

A systematic literature search was conducted with an expert librarian using keywords such as ‘digital scribe’, ‘large language model’, ‘generative AI’, and ‘patient-provider’. The full query is provided in the *Supplementary Materials*. Searches were performed in Embase, Web of Science, Medline, and Google Scholar on February 6, 2025, and included studies published up to that date. Peer-reviewed, original research articles in English were included. Preprints were included if published during the review process, which was concluded on February 28, 2025. Eligible studies focused on patient-provider conversations using transcription data and LLMs to generate clinical notes. Only empirical and implementation studies were considered, regardless of the development stage. Studies on other tasks, such as radiology reports or patient summaries, or using technologies other than LLMs, were excluded.

Two reviewers (EK and FN) independently performed title and abstract screening to identify eligible studies. Discrepancies were resolved through discussion until consensus was reached. Full texts were also independently assessed using predefined inclusion and exclusion criteria. No unresolved disagreements occurred; however, a third reviewer (MS) was designated to consult on any disputes if necessary. The screening and review process was managed using the Covidence platform.

### Data Collection and Extraction

From each study, the author, publication year, country, study name and design, population and demographics, ASR and LLM models with performance, validation methods and metrics, implementation phase, and results were extracted. As the included studies varied widely in their evaluation approaches, we explicitly distinguished validation by evaluation context. Technical validation is defined here as model‑level performance assessment using synthetic data, tested in a controlled setting. Clinical validation is defined as the evaluation of digital scribes used on recorded real-world consultations with or without live deployment. This distinction was used to support consistent interpretation of study maturity alongside TRL (Technology Readiness Level), which is described below.

### Technology Readiness Level

To assess the implementation maturity of digital scribes using LLMs and ASR, the TRL framework was applied. Originally developed by NASA, the 9-point scale evaluates the progression of a technology from basic research (TRL 1) to full integration and routine use (TRL 9). The adaptation by Fleuren et al. [10] was adopted, which tailors the TRL model to healthcare innovations. In this version, TRL levels 3 and 4 are combined into a single category, ‘Model prototyping and development’, to reflect the iterative nature of clinical tool design. A brief description of the adapted levels is provided in Supplementary Table A.

Each study was independently assessed by two reviewers (EK and FN) based on extracted information. Discrepancies were resolved through discussion. TRL assignment was based on the reported implementation phase, setting, and validation approach.

### Motivational Frameworks

To categorise the rationales behind developing and implementing AI-powered documentation tools in clinical settings as reported in the included studies, we developed a set of motivational frames. These frames were not derived from existing literature but were created inductively to group recurring themes observed across the included studies. They were defined as: human-oriented (focusing on user experience and clinician well-being), performance-oriented (focusing on documentation quality, efficiency, or safety) or system-oriented (focusing on the novelty and technical capabilities of the underlying AI systems). Identifying these frames provides a conceptual lens to interpret how validation methods are chosen, and which outcomes are prioritised, directly informing our analysis of evaluation practices across TRL stages.

### Data Availability

The data underlying this article can be shared on reasonable request to the corresponding authors.

## Results

**Fig. 1:**
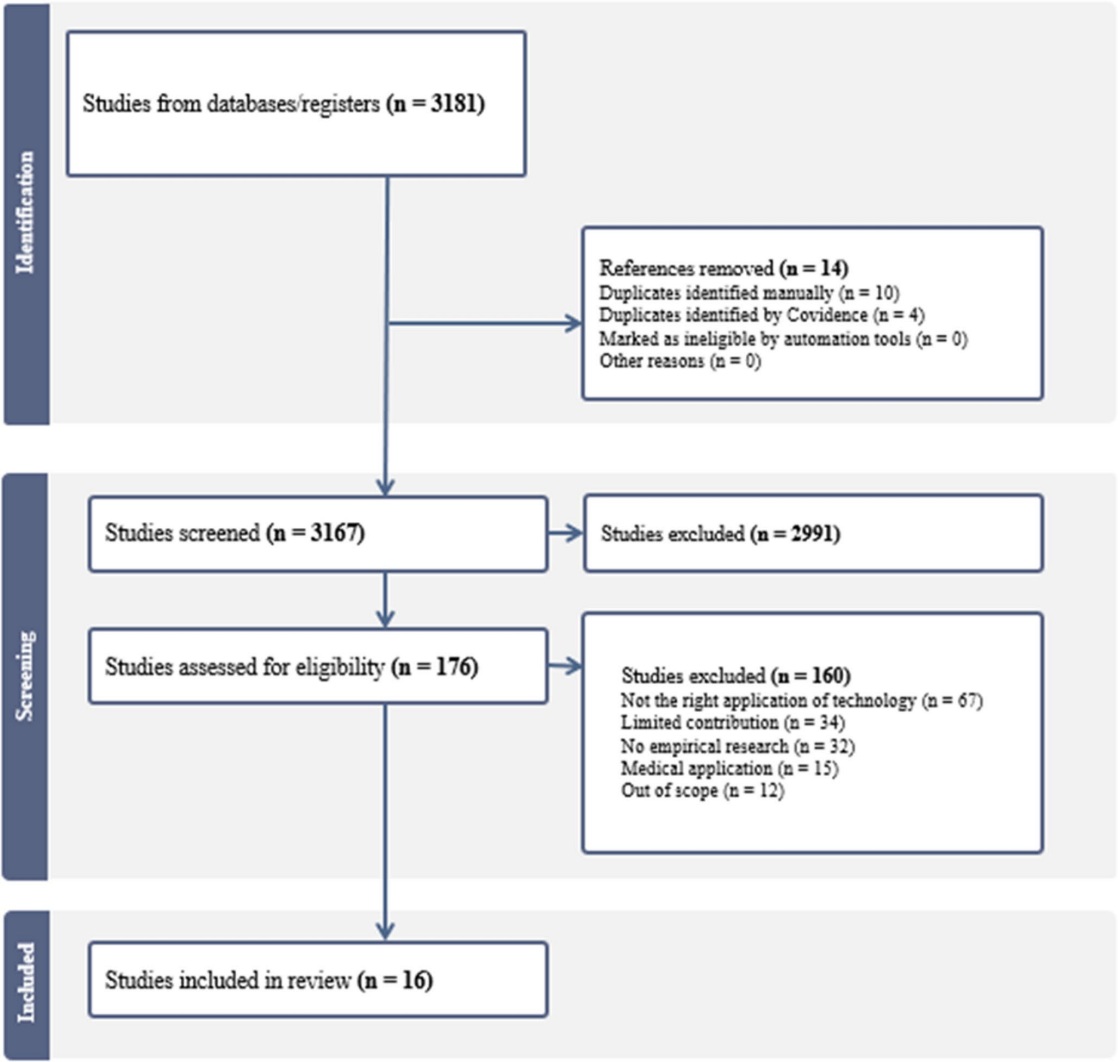
PRISMA diagram

The search yielded 3181 articles, with 176 remaining after title and abstract screening, and 16 included after full-text review (Fig. 1). Study details are available in Supplementary Table B.

The included studies, published between 2020 and 2025, were primarily conducted in the United States [3, 5, 11, 15, 18, 22], Australia [2, 13, 14], and the United Kingdom [17, 21]. Three used qualitative methods [3, 12, 16], nine used quantitative approaches [2, 5, 11, 13–15, 18, 21, 22], and the rest employed mixed or multi-methods designs [1, 17, 19, 20]. Seven studies used synthetic data or simulated conversations [1, 13–17, 21], five used real clinical datasets without live deployment [2, 18–20, 22], and four evaluated the technology in real-world clinical settings [3, 5, 11, 12].

Most studies used OpenAI’s GPT models for summarization [1, 3, 13–18, 21], while BART-based models appeared in fewer cases [12, 19, 22]. Transcription models were often unspecified [2, 13, 16–21]. Four studies described prompting strategies to guide the structure or content of AI-generated notes [3, 5, 12, 20].

### Validation Methods

To assess the performance, efficiency, accuracy, and consistency of digital scribes, various validation methods were used; however, none of the studies employed an established, published validation framework. The overview of the included studies and their validation method is included in Table 1. Comparative evaluation was used in ten studies, where clinician-written summaries are compared to automatically generated summaries [1, 2, 13–15, 17–19, 21, 22]. Three studies expanded this approach by including manually edited AI outputs as an intermediate step, reflecting how digital scribes are often used in clinical workflows: the LLM generates a draft, which the clinician reviews and modifies before entering it into the EHR [1, 11, 19]. This method provides a more realistic assessment of how digital scribes would perform in practice. Three studies used questionnaires to assess user experience and summary quality [5, 11, 17], two of these [5, 11] first provided pre-usage questionnaires, to compare clinician perceptions of administrative burden before and after implementation. Qualitative methods were used to assess user expectations and experience, either by interviews [1, 3, 12, 19, 20], focus groups [17] or user observations [12], to observe how clinicians interacted with AI-generated documentation in real time. None of the studies incorporated the patient perspective in their evaluation.

**Table 1:** Validation methods used in included studies, alphabetically ordered

Author (reference)	TRL	Technical/ Clinical	Qualitative/ quantitative	Validation method
Albrecht et al. [11]	7	Clinical	Quantitative	Pre- & post implementation survey
Balloch et al. [17]	3&4	Technical	Both	Comparative evaluation - manual vs. automated, User experience questionnaire & Focus group
Bundy et al. [3]	7	Clinical	Qualitative	User experience interview
Chen et al. [13]	3&4	Technical	Quantitative	Comparative evaluation - manual vs. automated
Fraile Navarro et al. [14]	5	Technical	Quantitative	Comparative evaluation - manual vs. automated
Galloway et al. [5]	7	Clinical	Quantitative	Pre- & post survey
Han et al. [20]	3&4	Technical	Both	Pre-usage interview, feedback study new method
Kaneda et al. [16]	3&4	Technical	Qualitative	N/A
Kernberg et al. [15]	3&4	Technical	Quantitative	Comparative evaluation - manual vs. automated
Knoll et al. [12]	6	Both	Qualitative	Pre-usage interview, User experience interview, User observations
Li et al. [19]	3&4	Technical	Both	Comparative evaluation - manual vs. automated
Moramarco et al. [21]	3&4	Technical	Quantitative	Comparative evaluation - manual vs. automated
Quiroz et al. [2]	2	Technical	Quantitative	Comparative evaluation - manual vs. automated
Sezgin et al. [22]	5	Technical	Quantitative	Comparative evaluation - manual vs. automated
Van Buchem et al. [1]	3&4	Technical	Both	Comparative evaluation - manual vs. automated, User experience interview
Van Veen et al. [18]	3&4	Technical	Quantitative	Comparative evaluation - manual vs. automated

#### Technical and Clinical Validation

Ten studies conducted a technical validation (Table 1), using synthetic transcripts or simulated conversations in a controlled setting [1, 12, 13, 15–21]. In these studies, documentation output was assessed by clinicians [12, 17, 19–21], medical students [1], or researchers [15, 16].

Three studies evaluated the systems using retrospective clinical data, based on recorded patient-provider conversations but without live deployment of the scribe in clinical workflows [2, 14, 22]. Four studies conducted a clinical validation study, using prospective testing in real-world clinical encounters, three as pilot implementations focused on feasibility [3, 5, 11], one as a single live test within a broader multi-phase protocol [12]. Two of these studies used pre- and post-usage surveys to evaluate user experience [5, 11], while the other two relied primarily on pre- and post-usage interviews [3, 12]. Across the included studies, explicit and systematic clinical validation methods, such as structured evaluation of accuracy, safety, or workflow impact during routine clinical use, were rare.

**Fig. 2:**
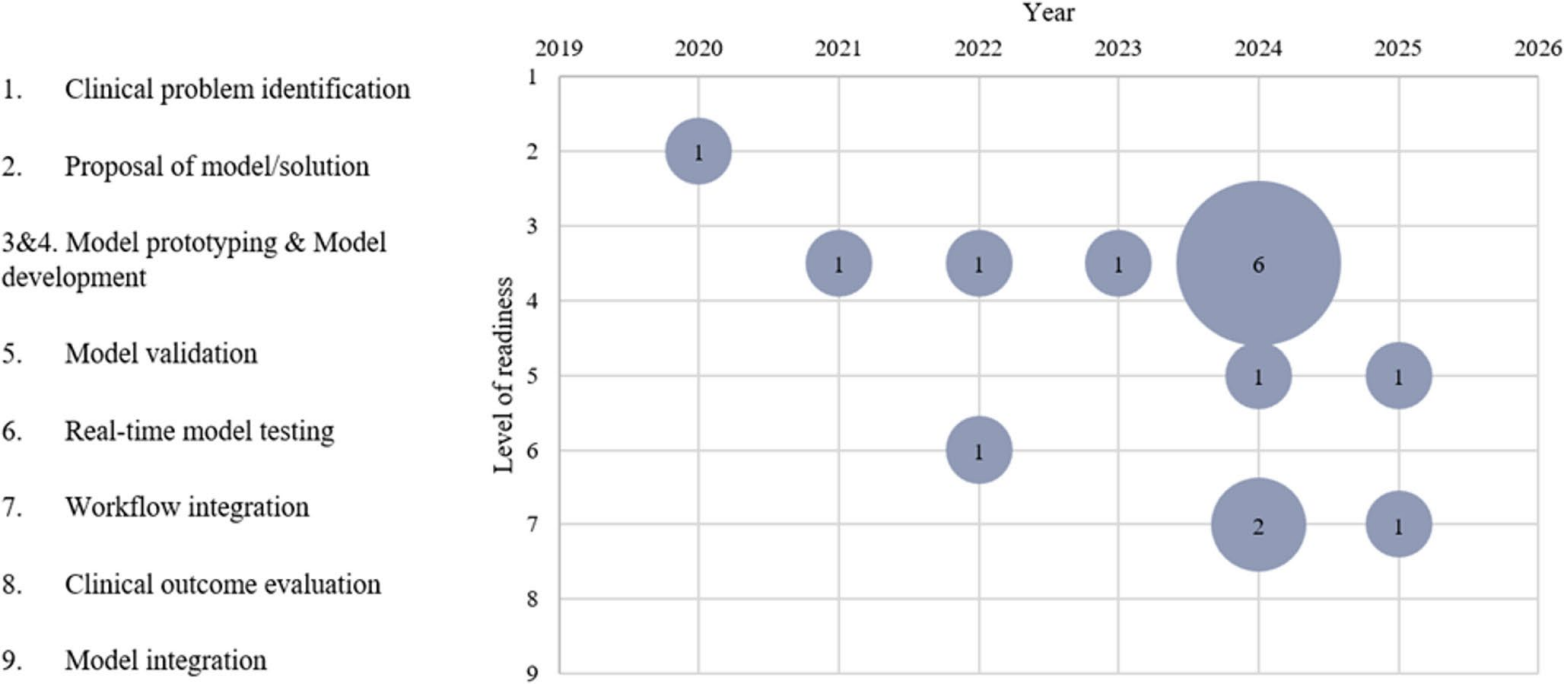
Technological readiness level (TRL)

The TRL of the digital scribe systems evaluated did not exceed workflow implementation [3, 5, 11] (Fig. 2). The lowest level observed in the studies was the proposal of model/solution [2], the second level on the technology readiness scale. Nine studies were situated at the stage of model prototyping & development (TRL 3&4) [1, 13, 15–21], while two studies performed model validation [14, 22] and real-time model testing was done in one study [12]. A modest progression toward higher TRLs was observed in more recent studies, although none reached full clinical deployment. A similar progression can be seen in the number of studies, increasing from one in 2020 to nine in 2024.

#### Evaluation Metrics

The evaluation of digital scribes varied considerably across studies, particularly in the quantitative metrics used to assess summary quality and performance. Justification for the choice of metrics was often lacking. When discussion of the used evaluation metrics was available, BertScore, METEOR, and Levenshtein distance were deemed most suitable for comparison of hand-written and generated notes, each capturing a complementary aspect of textual similarity [21]. Accordingly, although ROUGE is employed in seven studies as a measure of textual overlap, it is cited only once as less suitable in this context, given that it relies on lexical matching, and although LLMs may differ textually from the golden standard, can maintain a high summary quality [14]. Table 2 summarizes the four main types of validation metrics used.

**Table 2:** Validation metrics

Validation metrics	Quantitative metrics	Metric description
Text overlap measures	ROUGE [1, 13, 14, 18, 19, 20, 21, 22]; CHRF [21]; METEOR [13, 16, 21]; BLEU [13,16, 18, 21]; Completeness, Conciseness, Correctness [18]; Coherence, Consistency, Fluency, Relevance [14]; Quantitative text metrics (word count & lexical diversity) [1]; Flesch-Kincaid Grade Level [13]	Measure lexical similarity, linguistic quality, and text characteristics by matching strings at the character, word, or n-gram level
Edit distance metrics	Levenshtein edit distance, WER, MER, WIL [21]; Language use and precision, clarity and detail, coherence and flow, structural differences, stylistic variations, most common deletions & insertions [1]; Errors: Additions [15]; Errors: Incorrect statements, Omissions [15, 21]	Capture surface-level differences by counting character- or word-level transformations needed to convert system output to reference
Embedding based metrics	SkipThoughts, EmbeddingAverage, VectorExtrema, GreedyMatching, WMD, Moverscore [21]; BERTScore [1, 13, 16, 18, 20, 21]; UniEval [14]; Entity LINK – information accuracy CORRECT [22]	Assess semantic similarity by encoding text with pretrained models and computing cosine similarity in vector space
Fact extraction tools	Stanza, Snomed [21]; MEDCON [18]; PDQI-9 [1,15]; SAIL – Sheffield Assessment Instrument for Letters [17]; NASA Task Load Index – mental, physical, temporal demand, performance, effort, frustration [17]; Time spent on summary [1]	Evaluate factual accuracy by extracting entities or claims and comparing them between system output and reference text

### Framing and Reported Outcomes

The included studies revealed a range of reported outcomes and motivations for adopting digital scribes. As shown in Table 3, each study aligns with specific frames and themes, providing context for reported outcomes and adoption rationales across varied clinical environments.

**Table 3:** Motivational frames

Frame	Description	Themes
Human-oriented	Focuses on user experience, including clinician well-being and attentional presence	Reduced documentation time and administrative burden [1–21]
		Clinician engagement, burnout and work satisfaction [1–3, 5, 11–13, 16, 19–21]
Performance-oriented	Emphasizes tool effectiveness and measurable outcomes such as accuracy, efficiency, and clarity.	Potential for improved documentation quality and structure [1, 12–15, 17–21]
		Expected gains in safety or overall care quality [1–3, 11, 13, 15, 19, 21, 22]
		Enhanced workflow and efficiency [1–3, 5, 11, 12, 15, 17–21]
System-oriented	Highlights integration into workflows and technical novelty of the underlying AI tools.	Adaptation to existing digital workflows [1, 3, 5, 11, 12, 15, 19–21]
		Novelty and technical capabilities of tools [2, 13, 15, 16, 20–22]

#### Human-oriented

A consistent theme across the included studies is that digital scribes can meaningfully influence how clinicians experience patient encounters. 5 out of 16 included studies reported that automating notetaking enabled clinicians to shift their attention away from screens and administrative tasks and toward direct patient communication, reducing multitasking demands and improving the quality of interaction [3, 12, 16, 17, 21]. In research by Balloch et al. [17], simulated consultations with 8 clinicians showed a 23% reduction in screen time, and clinicians described being able to maintain attention more consistently. Knoll et al. [12] similarly reported that 78% of clinicians (*n* = 5) felt more present during patient interactions. In the Abridge implementation study [3], involving 93 pre‑implementation and 99 post‑implementation respondents, clinicians had 6.91 times higher odds of reporting easier documentation workflows, which they linked to improved attentional presence. By relieving clinicians from the repetitive nature of manual documentation, digital scribes helped reduce screen time and administrative overhead.

However, concerns about increased patient loads following the introduction of digital scribes were also expressed [3]. Although documentation time decreased, several felt pressured to use the saved time for additional patient visits, thereby increasing their overall workload [11, 15, 19]. Li et al. [19] supported this, noting that while fatigue scores dropped, indicating a 24% reduction, 12 physicians reported that time savings were offset by higher output expectations. This raised concerns about care quality, as LLM-generated summaries still required human review before finalization [19].

Many clinicians reported feeling more engaged and less fatigued during patient interactions [3, 5, 11, 12, 17, 19, 20]. Knoll et al. [12] showed a significant increase in usability scores (*P* < .05), and 78% of clinicians reported feeling more engaged during patient interactions. Balloch et al. [17] observed a 23% reduction in screen time, with 72% of clinicians reporting improved satisfaction, though statistical significance was not assessed. Five studies reported that clinicians felt more connected to their patients when the digital scribe managed documentation, allowing for less screen time and better direct communication [1, 2, 5, 19, 20]. In the research of Galloway et al. [5], with 117 onboarding and 55 follow‑up respondents, clinicians reported improved perceptions of documentation usability and reduced negative impact on well‑being after 60 days. However, experiences varied. While many clinicians reported greater engagement, others struggled with the tool’s format and limited expressiveness [11, 13]. Those who favoured freeform, narrative documentation, particularly in fields such as psychiatry and palliative care, found LLM summaries overly rigid and reductive, and though when GPT-4 performed well in palliative care consultations, it often failed to capture subtle interpersonal and emotional cues. These findings highlight how personal documentation preferences and specialty-specific demands shape adoption and perceived usefulness.

#### Performance-oriented

Most studies indicated that AI-driven documentation tools can improve documentation efficiency and quality [1, 3, 5, 11, 12, 17–20]. For example, van Buchem et al. [1] found that clinicians spent less time manually summarizing when using AI-assisted tools compared to traditional methods, a median of 186 s versus 202 s (*n* = 21 clinicians), respectively, indicating an average time reduction of 8%.

Similarly, Galloway et al. [5] reported that clinicians found LLM-generated notes easier to use, due to clear formatting and phrasing, leading to significantly improved ratings for clarity and ease of completion. In their sample of 117 onboarding respondents and 55 follow‑up respondents, positive ratings for note clarity and completeness increased from 41.9% to 71.0%, and ease‑of‑completion ratings rose from 32.3% to 48.4% (*p* < .05). Clinicians described this as a more structured workflow that reduced the need to reformat or interpret text, thereby speeding up the process of reviewing and updating notes. Findings across the included studies show a lot of variation in how useful and effective LLM-generated documentation is. These differences depend on medical specialty, how people normally write their notes, and what they expect a good note to look like. Knoll et al. [11] observed how clinicians worked with LLM-generated notes and found that those who often used structured formats, like SOAP notes or templates, got used to digital scribes more quickly. On the other hand, clinicians who were used to writing in freeform took longer to adjust, since the structured format didn’t match their preferred writing style. Similarly, Van Buchem et al. [1] found word choice and how summaries were built depended on the user’s specialty and expectations, which affected how useful the notes seemed.

#### System-oriented

Several studies focused their research on the novelty of AI technology in digital scribes and their need for workflow integration [3, 5, 13, 16]. Clinicians with long-standing documentation habits voiced that the mandatory use of digital scribes disrupted familiar routines and made adaptation challenging [1, 3, 12]. The shift to structured prompts or editing machine-generated notes conflicted with existing workflows, especially for those accustomed to composing freeform narratives [1, 12]. This friction affected how notes were reviewed, adjusted, and entered into the EHR system [1, 11, 12]. The studies collectively underscore that personal and professional preferences are central to successful integration of digital scribes into everyday practice.

Furthermore, perceived documentation quality was influenced by the complexity of source material. Kernberg et al. [15] demonstrated that longer and more intricate transcripts reduced the accuracy of AI-generated SOAP notes, with a mean consistency score of 52.9% and omissions accounting for 86% of errors. These results were statistically significant (*P* < .001), highlighting current limitations in processing detailed documentation. While the underlying technology presents clear innovations, these findings emphasize that the novelty of AI tools is still constrained by evolving technical boundaries, particularly in nuanced clinical scenarios.

Overall, the studies demonstrate that system‑level factors, such as EHR integration [3, 5], interface stability [11, 13], and alignment with existing documentation routines [1, 12], play a decisive role in adoption. When system components function reliably and fit established workflows, clinicians can incorporate digital scribes with minimal disruption. When they do not, trust, usability, and workflow integration are significantly hindered.

## Discussion

This scoping review examined how digital scribes that combine ASR and LLMs are validated, with a focus on their TRL and clinical performance. Across the included studies, three overarching themes emerged. By explicitly incorporating TRL as a lens for analysis, this study offers a novel perspective on how the maturity level of these technologies influences the choice and rigor of validation methods. First, validation methods varied widely and lacked standardization. While most studies relied on comparative evaluation, the metrics used differed substantially, limiting comparability. Second, the majority of studies assessed systems in synthetic or retrospective settings, with only four pilot studies evaluating digital scribes during live clinical use. As such, none progressed beyond workflow implementation at TRL 7, underscoring the early developmental stage of this technology. Third, reported benefits were often framed in terms of reduced administrative burden, improved documentation quality, or the perceived advantages of integrating LLMs, such as enhanced contextual understanding and more natural language generation. However, challenges such as hallucinations, omissions, and inconsistent performance remain unresolved. Taken together, these findings suggest that although digital scribes show clear potential to improve documentation efficiency and clinician satisfaction, robust real-world validation and standardized evaluation frameworks are urgently needed before widespread clinical adoption can be justified.

This review advances the field beyond earlier work, most notably the review by Van Buchem et al. [23], which focused primarily on ASR-based systems and transcription accuracy. It also extends recent contributions from Wang et al. [24], who systematically evaluated AI transcription tools by benchmarking ASR performance across clinical settings, highlighting variability in word error rates and note completeness. Similarly, Ng et al. [25] emphasized the challenges of adapting speech models to clinical environments and the inconsistency of word error rates across systems. While these prior reviews have provided valuable insights into ASR performance, they focused on transcription fidelity and technical limitations, either without examining how validation strategies evolve with technological maturity or without the integration of LLMs. By contrast, this study provides a systematic synthesis of LLM-enabled digital scribes, presenting validation methods and reported outcomes. Moreover, it applies a TRL framework specifically to LLM-based scribes, positioning these tools within the broader innovation pipeline. Finally, by introducing motivational framing, this review offers an interpretive perspective that situates acceptance and performance within the underlying rationales for adoption. Together, these contributions provide a timely overview of a rapidly evolving field and identify the methodological and clinical priorities needed to move digital scribes from experimental prototypes toward reliable clinical tools.

### Heterogeneity of Validation Methods

A key finding of this review is the striking absence of real-world validation studies for digital scribes. Despite growing interest and commercial deployment, most evaluations remain confined to controlled environments, with limited attention to clinical realism. Although technical validation supports early development by enabling consistent benchmarking under controlled conditions, it often overlooks the complexity of real-world settings, where patient variability, diverse documentation styles and workflow challenges are constant [26]. The reliance on shortened transcripts or structured summaries in some studies limits the representational fidelity of clinical consultations [14, 22], especially since transcript length is known to correlate with accuracy [15]. Evaluations conducted by medical students or researchers may also fall short, as they often lack the clinical judgment and workflow awareness of experienced practitioners. These limitations have broader implications for the perceived reliability and integration potential of digital scribes. Tools that perform well in controlled environments may fail to deliver meaningful outcomes in clinical use without robust, prospective trials, introducing inefficiencies such as extensive post-editing, negating their intended benefit. This is reflected in the low TRLs assigned to each study, which ranged from prototyping to limited workflow implementation, with no studies progressing to full-scale clinical outcome evaluation [3, 5, 11, 12]. Although there is a visible upward shift in TRL over time, it remains modest compared to the pace of real-world adoption, where commercial scribes are already being integrated into EHRs, implying the scribes currently in use have not been properly validated. The deployment of seemingly effective scribes without rigorous validation poses a significant concern, as it may lead to undetected errors or biases, risks introducing inaccuracies or inconsistencies into medical records and jeopardize patient safety.

### Current Metrics, Critique and Best Practices

Text-overlap metrics like ROUGE are widely used, yet their relevance to digital scribes is increasingly questioned. For instance, Fraile Navarro et al. [14] argue that ROUGE fails to capture semantic accuracy, especially in LLM-generated summaries that differ lexically but remain clinically valid. Li et al. [19] reported ROUGE-L scores of 0.30–0.40 alongside issues in factual consistency. Three years later, Han et al. [20] observed better usability and relevance, yet ROUGE scores remained similar, highlighting the metric’s insensitivity to qualitative gains. Other metrics such as BLEU, METEOR, BERTScore, and Levenshtein distance have also been used, though inconsistently and often without clear justification. In addition, these metrics often fail to capture improvements in semantic precision or clinical relevance. What may appear as stagnation in score-based assessments could mask meaningful gains in trustworthiness, clarity, and workflow alignment. This highlights the need to align evaluation methods with TRL stage, where early-phase systems may require different metrics than those approaching clinical implementation. While alternatives like completeness, conciseness, and correctness have been proposed [18], their inconsistent use and weak justification limit their utility. The variability in metrics and application makes it difficult to compare studies, assess generalizability, and identify top-performing approaches [3, 5, 11, 12]. Moreover, many studies rely on technically convenient metrics without clear rationale, raising concerns about clinical relevance and interpretability [3, 11].

However, a few studies stand out as examples of best practice. Moramarco et al. [21] employed one of the most comprehensive scoring mechanisms, incorporating and assessing four distinct categories of validation metrics. Similarly, Balloch et al. [17] and Knoll et al. [12] applied multi-phase validation frameworks that extend beyond technical benchmarking to include early usability testing and live clinical implementation. While most studies did not explain their choice of metrics, these examples demonstrate how structured, transparent methodologies can improve interpretability, support clinical relevance, and set a precedent for future evaluation standards. They illustrate how thoughtful validation design, not just metric selection, can strengthen credibility and comparability across the field and reinforce that it is not only the technology that evolves, but also the interpretive frameworks that shape perceptions of its success. Furthermore, they suggest that the main barriers to adoption lie not in the technology itself, but in workflow integration, clinician adaptation, and interface design.

Overall, these limitations of evaluation metrics and validation methods raise the question why approaches differ so distinctly across studies. Some rely on technical benchmarking under controlled conditions, others use retrospective comparisons or expert review, and only a few attempt prospective clinical trials. While LLMs represent a relatively new technology, several digital health validation frameworks already exist, and more have emerged post-LLM adoption, such as CONSORT-AI [27], DECIDE-AI [28] and TRIPOD-LLM [29]. Though not tailored to digital scribes, elements of these frameworks could have been adapted to this use case. Using such a standard approach could enhance consistency and accountability in validation by offering predefined criteria for study design, outcome selection, and reporting. For example, frameworks like CONSORT-AI encourage transparency in how AI outputs are generated and interpreted, while DECIDE-AI emphasizes early-stage clinical evaluation and human factors, and TRIPOD-LLM promotes rigorous reporting of model performance and generalizability. Applying these principles to digital scribes would help ensure that evaluations consistently address usability, factual accuracy, and clinical relevance, making results more comparable across studies and more actionable for implementation.

### Motivational Framing

This review introduces three motivational frames: human oriented, performance oriented, and system oriented, which shape how digital scribes are evaluated across the literature and across different stages of technological maturity. These frames do more than organise perspectives; they embed assumptions that influence which outcomes are prioritised and how technologies are interpreted in both research and clinical practice. The framing choices directly influence which success criteria are used, shaping study design, metric selection, and interpretation. For example, human-oriented studies may rely on subjective feedback or burnout metrics, while performance-oriented studies favour structured usability testing. Each frame carries distinct expectations of ‘success’. While perceived human-oriented benefits, such as reduced documentation burden and improved clinician wellbeing, are often highlighted, their sustainability across roles, specialties, and documentation styles remains unclear [5, 11]. This variability underscores the need for adaptable and customizable tools that can accommodate diverse clinical contexts and preferences. Reported relief in burden may be uneven, and work pressure could be redistributed into other tasks and challenges [1, 21]. Notably, many of these studies were conducted at early TRLs, where user feedback is based on prototypes or short-term exposure, limiting insight into long-term impact.

The performance-oriented frame often portrays a purely metric-driven approach, which may overlook the nuanced, relational aspects of clinical work. Increased speed and precision do not necessarily capture the interpretive demands of documentation [2, 15]. This is especially true for systems at TRL 3–5, where evaluation focuses on technical benchmarks rather than clinical integration.

In contrast, the system-oriented frame tends to emphasize technological advancement as a goal in itself, often prioritizing innovation milestones over clinical utility. Here, success is measured by system capabilities, such as model size, integration speed, or novelty, rather than how well the technology supports clinicians in practice. This disconnect between innovation and clinical applicability is especially visible in studies where low-TRL systems are presented as ready for use, despite persistent issues such as hallucinations, transcription errors, and contextual inaccuracies. Without grounding innovation in real-world utility, digital scribes risk underdelivering in practice, despite promising results in controlled settings. Premature implementation can lead to misalignment with clinical workflows, limited uptake by end users, and inefficiencies such as extensive post-editing that negate the intended benefit [3, 21].

Ultimately, motivational frames can influence how digital scribes are evaluated. By recognising how framing shapes evaluation practice, researchers and developers can better align their methods with clinical realities and stakeholder needs. Making these frames explicit helps avoid skewed expectations, improves comparability across studies, and supports more responsible implementation.

### Future Work

Given the rapid advancement of LLM and ASR technologies, future research should prioritise the development of a robust validation framework tailored to AI-generated clinical documentation. This framework should assess reliability, relevance, and clinical utility, incorporating clinical endpoints and measures for semantic accuracy, usability, and long-term integration. The selection of evaluation metrics must be aligned with both the motivational framing and the maturity stage (TRL) of the tool, as these factors shape what is measured, how results are interpreted, and which outcomes are prioritised. The three motivational frames (human-, performance-, and system-oriented) reflect the underlying goals and perspectives that shape evaluation priorities and can guide what such a framework should include. Existing frameworks for AI in healthcare offer a foundation but require adaptation to address the dual role of digital scribes in transcription and summarization. To ensure safe and effective adoption, future frameworks must shift from technical metrics toward clinically meaningful outcomes, such as efficiency, safety, communication quality, and documentation burden. Incorporating criteria for note quality, workflow compatibility, and safety would improve consistency and enable meaningful cross-study comparisons.

Real-world validation remains a critical gap. Future studies should move beyond technical benchmarking and evaluate clinical effectiveness through pilot programmes, longitudinal deployments, and multi-centre or multi-national trials, to scale TRLs and evaluate digital scribes in real-world clinical settings. This would allow assessment across diverse clinical contexts and clarify whether early usability gains translate into sustained improvements in care quality and clinician experience [30]. To support this, reproducibility also demands attention. Limited transparency around ASR models and prompt engineering hinders replication and undermines clinical trust. As Bundy et al. [3] note, transcription quality and prompt design significantly shape output, yet most studies lack sufficient methodological detail. Finally, expanding geographical and linguistic diversity is essential. Many regions, especially non-English healthcare systems, remain underrepresented. Including diverse contexts will deepen understanding of cultural, regulatory, and infrastructural factors influencing adoption. Given the pace of innovation, maintaining a living review or regularly updating existing ones will help keep findings relevant and actionable. Ultimately, safe clinical adoption demands validation efforts that are as rigorous and rapid as the technologies themselves.

Multiple analytical perspectives can be applied when expanding research on digital scribes. In particular, regulatory, economic, and ecological dimensions should be taken into account for comprehensive evaluation. Under the European Union Medical Device Regulation (EU MDR), classification of digital scribes may depend on their intended purpose and whether their outputs are used to inform clinical decisions. There is substantial debate whether digital scribes drive or influence medical decisions and should therefore be classified as a medical device under the European Union Medical Device Regulation [31]. This classification carries substantial implications, including requirements for clinical evaluation, risk management, and post‑market surveillance that current validation practices rarely satisfy. Regulatory approaches also differ between jurisdictions; in the United States, certain low-risk, clinician-supervised AI tools used for documentation or administrative support may be subject to different regulatory treatment and, in some cases, may not be actively enforced as medical devices [32]. Meanwhile, the NHS England guidance indicates consultation-summarisation tools may qualify as medical devices depending on their intended use and require all vendors to hold at minimum MHRA Class 1 registration [33]. This underscores the need for future research to explore how evolving regulatory frameworks influence validation standards, evidence requirements, and safe integration into clinical practice, especially considering future expansion of digital scribe functionalities. From an economic perspective, largescale language models require substantial investment for licensing, integration, and maintenance, yet none of the included studies reported cost or resource requirements. Evaluations should therefore include cost-effectiveness analyses, return on investment modelling, and assessments of long-term operational costs. In parallel, the energy demands of LLMs raise questions about environmental sustainability. Research should quantify the ecological footprint of digital scribes and determine whether the time savings they offer justify their financial and environmental costs, supporting more responsible and sustainable implementation.

Lastly, the dimension that requires separate consideration is the patient perspective [34]. Although digital scribes are frequently presented as improving patient–clinician communication, patients themselves are largely absent from existing studies, with no included study assessing patient reported outcomes, satisfaction, or perceptions of accuracy. As digital scribes increasingly mediate the documentation of clinical encounters, future work should examine how they affect communication quality, patient understanding, and perceived safety, as well as potential risks such as misrepresentation or omissions.

### Limitations

The field of AI-assisted clinical documentation is evolving rapidly, with new models and validation approaches emerging continuously. As a result, some of the studies included in this review, particularly those conducted prior to the release of GPT-4 or similar LLMs, may already be outdated or superseded by newer systems. To ensure relevance, peer-reviewed preprints published during the review process were also included. Nonetheless, as model capabilities continue to advance, the performance and applicability of digital scribes will likely improve further. However, the underlying validation frameworks, evaluation metrics, and implementation challenges identified in these studies remain pertinent and applicable to future developments.

Finally, while ASR and digital scribe tools are increasingly adopted in healthcare systems worldwide, this review was restricted to English-language publications due to resource constraints. As a result, research from non-English speaking regions, where different implementation strategies, validation standards, or cultural considerations may apply, could not be included. This may limit the global generalizability of the findings.

## Conclusion

This scoping review examined the validation methods and clinical readiness of digital scribes combining ASR and LLMs. Across the included studies, evaluation methods were highly heterogeneous and rarely aligned with established frameworks, with most relying on simulated or retrospective data. Only a small number progressed to limited, pilot‑scale clinical implementation, corresponding to early TRL stages (TRL 3&4). Reported benefits clustered around human‑, performance‑, and system‑oriented frames, yet these outcomes were often anticipated rather than consistently demonstrated through systematic clinical validation. Despite this early maturity, digital scribes are already being deployed in practice, creating a widening gap between commercial adoption and scientific evidence. Bridging this gap will require prospective, real-world studies that assess not only technical accuracy but also clinical outcomes, usability, and workflow integration. Developing robust, standardised validation frameworks tailored to digital scribes will be essential to support safe, effective, and evidence‑based integration into clinical care.

## Supplementary Information

Below is the link to the electronic supplementary material.

Supplementary File 1 (DOCX 106 KB)

## Data Availability

The data underlying this article can be shared on reasonable request to the corresponding authors.

## Declarations

RB received an independent research grant from Abbott to the institute and speaker fees from Abbott, AstraZeneca Bayer, Boehringer Ingelheim, and Novartis outside the scope of the submitted work.

Generative AI was used for formatting the reference list, AI output has been manually checked.
