# Supplementary material for: Validating Digital Scribes: A Scoping Review of Evaluation Practices and Clinical Use
Source: J Med Syst. 2026 Apr 24;50(1):62. doi: 10.1007/s10916-026-02392-3 (PMC13106249; doi:10.1007/s10916-026-02392-3)
Supplement: Supplementary file 1 — (DOCX 106 KB) [file 10916_2026_2392_MOESM1_ESM.docx]

**Supplementary Materials**

**Search string**

| **Database searched** | **Platform** | **Years of coverage** | **Records** | **Records after duplicates removed** |
| --- | --- | --- | --- | --- |
| Medline ALL | Ovid | 1946 - Present | 1088 | 1065 |
| Embase | Embase.com | 1971 - Present | 2454 | 1624 |
| Web of Science Core Collection* | Web of Knowledge | 1975 - Present | 1298 | 380 |
| Additional Search Engines: Google Scholar** | | | 200 | 112 |
| **Total** | | | **5040** | **3181** |

*Science Citation Index Expanded (1975-present); Social Sciences Citation Index (1975-present); Arts & Humanities Citation Index (1975-present); Conference Proceedings Citation Index- Science (1990-present); Conference Proceedings Citation Index- Social Science & Humanities (1990-present); Emerging Sources Citation Index (2005-present)

**Google Scholar was searched via "Publish or Perish" to download the results in EndNote.

No other database limits were used than those specified in the search strategies

**Medline**

(Artificial Intelligence/ OR Ambient Intelligence/ OR Machine Learning/ OR Deep Learning/ OR Natural Language Processing/ OR ((artificial* ADJ3 intelligen*) OR ((machine OR deep) ADJ learning) OR ((software* OR automat*) ADJ3 (language* OR speech* OR voice*) ADJ3 recognition) OR (large* ADJ3 language* ADJ3 model*) OR (generative* ADJ3 (pretrain* OR pre-train*) ADJ3 transformer*) OR ChatGPT OR LLM OR chatbot* OR chat-bot* OR GPT OR (natural* ADJ3 (language OR speech OR voice*) ADJ3 processing*) OR ((speech OR language OR voice*) ADJ3 (technolog* OR recognition* OR software* OR machine-interpret*)) OR ((ambient* OR machine-interpret*) ADJ3 listening) OR (intelligent* ADJ3 listening ADJ3 framework*) OR generative-model* OR digital-scribe* OR autoscribe* OR auto-scribe*).ab,ti,kw. OR (ai).ti.) AND (((* Professional-Patient Relations OR Practice Patterns, Physicians'/ OR * Physicians/ OR * Patients/ OR (Patients / AND exp Health Personnel /)) AND (Communication / )) OR * "Referral and Consultation"/ OR (((doctor* OR provider* OR physician* OR clinician* OR professional* OR surgeon* OR nurse* OR practitioner) ADJ3 (patient* OR outpatient*) ADJ6 (communicat* OR conversation* OR dialog* OR interview* OR consultation* OR talk* OR encounter* OR audio* OR recording* OR administration* OR listening OR speech OR speaking OR summar*)) OR ((clinical*-practice*) ADJ6 (communicat* OR conversation* OR interview* OR consultation*)) OR (assist* ADJ3 (clinical* OR patient*) ADJ3 documentation*)).ab,ti,kw. OR (consultation* OR ((patient* OR outpatient*) ADJ6 (communicat* OR conversation* OR dialog* OR interview* OR consultation* OR talk* OR encounter* OR audio* OR recording* OR administration* OR listening)) OR ((clinical* OR patient*) ADJ3 (documentation* OR summar* OR companion*))).ti.) NOT (case report/ OR case-report*.ti.) AND english.la.

(* Large Language Models / OR * Natural Language Processing / OR (((software* OR automat*) ADJ3 (language* OR speech* OR voice*) ADJ3 recognition) OR (large* ADJ3 language* ADJ3 model*) OR language-model* OR (generative* ADJ3 (pretrain* OR pre-train*) ADJ3 transformer*) OR ChatGPT OR LLM OR chatbot* OR chat-bot* OR GPT OR (natural* ADJ3 (language OR speech OR voice*) ADJ3 processing*) OR ((speech OR language OR voice*) ADJ3 (technolog* OR recognition* OR software* OR machine-interpret*)) OR ((ambient* OR machine-interpret*) ADJ3 listening) OR (intelligent* ADJ3 listening ADJ3 framework*) OR generative-model* OR digital-scribe* OR autoscribe* OR auto-scribe*).ti.) AND (* Practice Patterns, Physicians' / OR ((* Validation Study / OR * Data Accuracy /) AND (Practice Patterns, Physicians' /)) OR (((clinical* OR medic* OR health* OR care* OR doctor* OR physician* OR oncolog* OR orthopaed* OR orthoped* OR ophthalm* OR anesth* OR anaesth* OR nurs* OR psychiatr* OR neuro* OR urolog* OR pharmac* OR dermatolog* OR gynecol* OR gynaecol* OR gp OR general-pract* OR surg*) ADJ3 (practice* OR office* OR communicat* OR interact* OR setting*)) OR ((validation* OR accura*) AND (healthcare OR health-care))).ti.)

**Embase**

('artificial intelligence'/de OR 'generative artificial intelligence'/exp OR 'ambient intelligence'/de OR 'artificial general intelligence'/de OR 'machine learning'/de OR 'deep learning'/de OR 'automatic speech recognition'/de OR 'large language model'/exp OR 'natural language processing'/de OR 'audio speech recognition'/de OR 'speech discrimination'/de OR ((artificial* NEAR/3 intelligen*) OR ((machine OR deep) NEXT/1 learning) OR ((software* OR automat*) NEAR/3 (language* OR speech* OR voice*) NEAR/3 recognition) OR (large* NEAR/3 language* NEAR/3 model*) OR (generative* NEAR/3 (pretrain* OR pre-train*) NEAR/3 transformer*) OR ChatGPT OR LLM OR chatbot* OR chat-bot* OR GPT OR (natural* NEAR/3 (language OR speech OR voice*) NEAR/3 processing*) OR ((speech OR language OR voice*) NEAR/3 (technolog* OR recognition* OR software* OR machine-interpret*)) OR ((ambient* OR machine-interpret*) NEAR/3 listening) OR (intelligent* NEAR/3 listening NEAR/3 framework*) OR generative-model* OR digital-scribe* OR autoscribe* OR auto-scribe*):ab,ti,kw OR (ai):ti) AND ('doctor patient communication'/de OR 'professional-patient relationship'/exp/mj OR (('professional-patient relationship'/exp OR 'clinical practice'/de OR physician/mj OR patient/mj OR (patient/exp AND ('health care personnel'/exp))) AND ('interpersonal communication'/de OR 'verbal communication'/exp OR conversation/de)) OR consultation/exp/mj OR (((doctor* OR provider* OR physician* OR clinician* OR professional* OR surgeon* OR nurse* OR practitioner) NEAR/3 (patient* OR outpatient*) NEAR/6 (communicat* OR conversation* OR dialog* OR interview* OR consultation* OR talk* OR encounter* OR audio* OR recording* OR administration* OR listening OR speech OR speaking OR summar*)) OR ((clinical*-practice*) NEAR/6 (communicat* OR conversation* OR interview* OR consultation*)) OR (assist* NEAR/3 (clinical* OR patient*) NEAR/3 documentation*)):Ab,ti,kw OR (consultation* OR ((patient* OR outpatient*) NEAR/6 (communicat* OR conversation* OR dialog* OR interview* OR consultation* OR talk* OR encounter* OR audio* OR recording* OR administration* OR listening)) OR ((clinical* OR patient*) NEAR/3 (documentation* OR summar* OR companion*))):ti) NOT ('case report'/de OR case-report*:ti) AND [english]/lim

('automatic speech recognition'/mj OR 'large language model'/exp/mj OR 'natural language processing'/mj OR 'audio speech recognition'/mj OR (((software* OR automat*) NEAR/3 (language* OR speech* OR voice*) NEAR/3 recognition) OR (large* NEAR/3 language* NEAR/3 model*) OR language-model* OR (generative* NEAR/3 (pretrain* OR pre-train*) NEAR/3 transformer*) OR ChatGPT OR LLM OR chatbot* OR chat-bot* OR GPT OR (natural* NEAR/3 (language OR speech OR voice*) NEAR/3 processing*) OR ((speech OR language OR voice*) NEAR/3 (technolog* OR recognition* OR software* OR machine-interpret*)) OR ((ambient* OR machine-interpret*) NEAR/3 listening) OR (intelligent* NEAR/3 listening NEAR/3 framework*) OR generative-model* OR digital-scribe* OR autoscribe* OR auto-scribe*):ti) AND ('clinical practice'/mj OR (('validation process'/mj OR 'data accuracy'/mj) AND ('clinical practice'/de)) OR (((clinical* OR medic* OR health* OR care* OR doctor* OR physician* OR oncolog* OR orthopaed* OR orthoped* OR ophthalm* OR anesth* OR anaesth* OR nurs* OR psychiatr* OR neuro* OR urolog* OR pharmac* OR dermatolog* OR gynecol* OR gynaecol* OR gp OR general-pract* OR surg*) NEAR/3 (practice* OR office* OR communicat* OR interact* OR setting*)) OR ((validation* OR accura*) AND (healthcare OR health-care))):ti)

**Web of science**

(TS=((artificial* NEAR/2 intelligen*) OR ((machine OR deep) NEAR/1 learning) OR ((software* OR automat*) NEAR/2 (language* OR speech* OR voice*) NEAR/2 recognition) OR (large* NEAR/2 language* NEAR/2 model*) OR (generative* NEAR/2 (pretrain* OR pre-train*) NEAR/2 transformer*) OR ChatGPT OR LLM OR chatbot* OR chat-bot* OR GPT OR (natural* NEAR/2 (language OR speech OR voice*) NEAR/2 processing*) OR ((speech OR language OR voice*) NEAR/2 (technolog* OR recognition* OR software* OR machine-interpret*)) OR ((ambient* OR machine-interpret*) NEAR/2 listening) OR (intelligent* NEAR/2 listening NEAR/2 framework*) OR generative-model* OR digital-scribe* OR autoscribe* OR auto-scribe*) OR TI=(ai)) AND (TS=(((doctor* OR provider* OR physician* OR clinician* OR professional* OR surgeon* OR nurse* OR practitioner) NEAR/2 (patient* OR outpatient*) NEAR/5 (communicat* OR conversation* OR dialog* OR interview* OR consultation* OR talk* OR encounter* OR audio* OR recording* OR administration* OR listening OR speech OR speaking OR summar*)) OR ((clinical*-practice*) NEAR/5 (communicat* OR conversation* OR interview* OR consultation*)) OR (assist* NEAR/2 (clinical* OR patient*) NEAR/2 documentation*)) OR TI=(consultation* OR ((patient* OR outpatient*) NEAR/5 (communicat* OR conversation* OR dialog* OR interview* OR consultation* OR talk* OR encounter* OR audio* OR recording* OR administration* OR listening)) OR ((clinical* OR patient*) NEAR/2 (documentation* OR summar* OR companion*)))) NOT TI=(case-report*) AND LA=(English)

(TI=(((software* OR automat*) NEAR/2 (language* OR speech* OR voice*) NEAR/2 recognition) OR (large* NEAR/2 language* NEAR/2 model*) OR language-model* OR (generative* NEAR/2 (pretrain* OR pre-train*) NEAR/2 transformer*) OR ChatGPT OR LLM OR chatbot* OR chat-bot* OR GPT OR (natural* NEAR/2 (language OR speech OR voice*) NEAR/2 processing*) OR ((speech OR language OR voice*) NEAR/2 (technolog* OR recognition* OR software* OR machine-interpret*)) OR ((ambient* OR machine-interpret*) NEAR/2 listening) OR (intelligent* NEAR/2 listening NEAR/2 framework*) OR generative-model* OR digital-scribe* OR autoscribe* OR auto-scribe*)) AND (TI=(((clinical* OR medic* OR health* OR care* OR doctor* OR physician* OR oncolog* OR orthopaed* OR orthoped* OR ophthalm* OR anesth* OR anaesth* OR nurs* OR psychiatr* OR neuro* OR urolog* OR pharmac* OR dermatolog* OR gynecol* OR gynaecol* OR gp OR general-pract* OR surg*) NEAR/2 (practice* OR office* OR communicat* OR interact* OR setting*)) OR ((validation* OR accura*) AND (healthcare OR health-care)))) AND LA=(English)

**Google scholar**

'artificial intelligence'|'machine|deep learning'|'automated language|speech|voice recognition|processing'|'language model'|chatgpt|'digital scribe' doctor|physician|clinician patient|patients communication|conversation|consultation

'automated language|speech|voice recognition|processing'|'large language model'|ChatGPT|'digital-scribe'|autoscribe 'clinical|medical|healthcare|doctor|physician practice|office'

**Supplementary Table A – Technology readiness levels**

| **TRL** | **NASA Definition** | **Definition by Fleuren et al. (2020) - Clinical definition** | **Application to digital scribes** |
| --- | --- | --- | --- |
| 1 | Basic principles observed and reported | Clinical problem identification | A digital scribe concept is identified to address a clinical documentation challenge. Literature is reviewed, and a research question is formulated. |
| 2 | Technology concept and/or application formulated | Proposal of model/solution | A digital scribe solution is proposed; relevant datasets and outcome measures are identified. |
| 3 | Analytical and experimental critical function and/or characteristic proof-of-concept | Model prototyping & Model development | A prototype is developed and initially validated on characterized clinical data to assess transcription and decision-support potential. |
| 4 | Component and/or breadboard validation in laboratory environment |  |  |
| 5 | Component and/or breadboard validation in relevant environment | Model validation | The model is validated on a representative dataset distinct from the training population, with standardized preprocessing pipelines. |
| 6 | System/subsystem model or prototype demonstration in a relevant environment | Real-time model testing | The model is tested in real-time and integrated into the EHR, without exposing outputs to clinical staff. |
| 7 | System prototype demonstration in an operational environment | Workflow implementation | The model is implemented in clinical workflow; outputs are shown to staff, and performance, safety, and usability are assessed. |
| 8 | Actual system completed and qualified through test and demonstration | Clinical outcome evaluation | The model is evaluated in a Phase 3 study, with outcomes assessed for effectiveness, safety, and care impact. |
| 9 | Actual system proven through successful mission operations | Model integration | The final model is deployed in clinical workflows and evaluated across sites to confirm it meets specifications. |

**Supplementary Table B – Data table**

| **Title** | **Author** | **Year** | **Country** | **Validation method** | **Qualitative/** **quantitative** | **Motivation (i.e. patient safety/quality/time savings)** |
| --- | --- | --- | --- | --- | --- | --- |
| Adapted large language models can outperform medical experts in clinical text summarization | van Veen, D.; van Uden, C.; Blankemeier, L.; Delbrouck, J. B.; Aali, A.; Bluethgen, C.; Pareek, A.; Polacin, M.; Reis, E. P.; Seehofnerova, A.; Rohatgi, N.; Hosamani, P.; Collins, W.; Ahuja, N.; Langlotz, C. P.; Hom, J.; Gatidis, S.; Pauly, J.; Chaudhari, A. S. | 2024 | USA | Comparative evaluation - current method (manual) vs new method (automatic) | Quantitative | Improved quality in summaries, saving time in answering clinical questions |
| AscleAI: A LLM-based Clinical Note Management System for Enhancing Clinician Productivity | Han, J.; Park, J.; Huh, J.; Oh, U.; Do, J.; Kim, D. | 2024 | South-Korea | Pre-usage interview, feedback study new method | Both | Omission of patient information, creation of inaccurate records, lower productivity, increased workload, variability in notes which complicates review process & extracting information, patient safety, clinical efficiency, physician time savings |
| Automating Clinical Documentation with Digital Scribes: Understanding the Impact on Physicians | Li, B.; Crampton, N.; Yeates, T.; Xia, Y.; Tian, X.; Truong, K. | 2021 | Canada | Comparative evaluation - current method (manual) vs new method (automatic), Interviews | Both | Documentation burden, Patient safety, physician time savings, documentation quality |
| Can the Administrative Loads of Physicians be Alleviated by AI-Facilitated Clinical Documentation? | Bundy, H.; Gerhart, J.; Baek, S.; Connor, C. D.; Isreal, M.; Dharod, A.; Stephens, C.; Liu, T. L.; Hetherington, T.; Cleveland, J. | 2024 | USA | User- experience interview | Qualitative | Reduced documentation time, better patient engagement, reduced cognitive burden, streamline workflow, increase accuracy |
| ChatGPT's potential in enhancing physician efficiency: a Japanese case study | Kaneda, Y.; Takita, M.; Hamaki, T.; Ozaki, A.; Tanimoto, T. | 2023 | Japan | n/a | Qualitative | Focused around chatgpt |
| Enhancing Clinical Documentation Workflow with Ambient Artificial Intelligence: Clinician Perspectives on Work Burden, Burnout, and Job Satisfaction | Albrecht, M.; Shanks, D.; Shah, T.; Hudson, T.; Thompson, J.; Filardi, T.; Wright, K.; Ator, G. A.; Smith, T. R. | 2025 | USA | Pre- & post implementation survey | Quantitative | Burnout & administrative burden |
| Evaluation of a Digital Scribe: Conversation Summarization for Emergency Department Consultation Calls | Sezgin, E.; Sirrianni, J.W.; Kranz, K. | 2024 | USA | Comparative evaluation - current method (manual) vs new method (automatic) | Quantitative | Burnout reduction, documentation burden, patient safety, efficiency, quality |
| Expert evaluation of large language models for clinical dialogue summarization | Fraile Navarro, D.; Coiera, E.; Hambly, T. W.; Triplett, Z.; Asif, N.; Susanto, A.; Chowdhury, A.; Azcoaga Lorenzo, A.; Dras, M.; Berkovsky, S. | 2025 | Australia | Comparative evaluation - current method (manual) vs new method (automatic) | Quantitative | Administrative burden & burnout |
| Exploring the opportunities of large language models for summarizing palliative care consultations: A pilot comparative study | Chen, X.; Zhou, W.; Hoda, R.; Li, A.; Bain, C.; Poon, P. | 2024 | Australia | Comparative evaluation - current method (manual) vs new method (automatic) | Quantitative | Development LLM - technical aspects |
| Human Evaluation and Correlation with Automatic Metrics in Consultation Note Generation | Moramarco, F.; Korfiatis, A. P.; Perera, M.; Juric, D.; Flann, J.; Reiter, E.; Belz, A.; Savkov, A. | 2022 | UK | Comparative evaluation - current method (manual) vs new method (automatic) | Quantitative | Dialogue quality, naturalness, coherence |
| Identifying relevant information in medical conversations to summarize a clinician-patient encounter | Quiroz, J. C.; Laranjo, L.; Kocaballi, A. B.; Briatore, A.; Berkovsky, S.; Rezazadegan, D.; Coiera, E. | 2020 | Australia | Comparative evaluation - current method (manual) vs new method (automatic) | Quantitative | Clinician burnout, time savings, documentation burden, EHR inefficiencies, and potential patient care disruption |
| Impact of a Digital Scribe System on Clinical Documentation Time and Quality: Usability Study | van Buchem, M. M.; Kant, I. M. J.; King, L.; Kazmaier, J.; Steyerberg, E. W.; Bauer, M. P. | 2024 | Netherlands | Comparative evaluation - current method (manual) vs new method (automatic), user experience interview | Both | Physician burnout & decreased work satisfaction |
| Impact of an Artificial Intelligence-Based Solution on Clinicians' Clinical Documentation Experience: Initial Findings Using Ambient Listening Technology | Galloway, J. L.; Munroe, D.; Vohra-Khullar, P. D.; Holland, C.; Solis, M. A.; Moore, M. A.; Dbouk, R. H. | 2024 | USA | Pre- & post survey | Quantitative | Clinician burnout, EHR burden, and potential workflow improvements |
| Use of an ambient artificial intelligence tool to improve quality of clinical documentation | Balloch, J.; Sridharan, S.; Oldham, G.; Wray, J.; Gough, P.; Robinson, R.; Sebire, N. J.; Khalil, S.; Asgari, E.; Tan, C.; Taylor, A.; Pimenta, D. | 2024 | UK | Comparative evaluation - current method (manual) vs new method (automatic), user experience questionnaire & focus group | Both | Increased workload, cognitive load, physician burnout |
| User-Driven Research of Medical Note Generation Software | Knoll, T.; Moramarco, F.; Korfiatis, A. P.; Young, R.; Ruffini, C.; Perera, M.; Perstl, C.; Reiter, E.; Belz, A.; Savkov, A. | 2022 | Dublin | Pre-usage interview, user experience interview, user observations | Qualitative | Addressing clinician burnout, better medical outcomes, and the need for detailed records |
| Using ChatGPT-4 to Create Structured Medical Notes From Audio Recordings of Physician-Patient Encounters: Comparative Study | Kernberg, A.; Gold, J. A.; Mohan, V. | 2024 | USA | Comparative evaluation - current method (manual) vs new method (automatic) | Quantitative | Importance of medical documentation & possible inaccuracies, physician burnout |
|  |  |  |  |  |  |  |
|  |  |  |  |  |  |  |

| **Evaluation metrics/indicators used** | **TRL** | **Aim/ purpose** | **Research design** | **Methods** | **Datasource** | **Technical/ Clinical** |
| --- | --- | --- | --- | --- | --- | --- |
| Correctness, completeness and conciseness- ROUGE, BLEU, BERTScore, MEDCON | Model prototyping & Model development | To assess if adapted LLMs can outperform specialists in answering real-world clinical questions | Comparative analysis | Fine-tuned models compared to specialists using NLP metrics, clinician reader study (10 physicians), and safety analysis for medical harm/fabrication. | Internal medical QA dataset adapted from clinical practice questions. | Technical |
| ROUGE, BERTScore, human rating on accuracy, coherence, usefulness | Model prototyping & Model development | To support physicians in efficiently managing clinical notes via LLM-based tools | Interviews + design probe with 6 clinicians; empirical dataset evaluation | System design tested with retrieval (BM25 + LLM reranker), summarization, and clustering. | MIMIC-III | Technical |
| ROUGE-L and human evaluation for factual consistency and completeness | Model prototyping & Model development | Automate clinical documentation (SOAP notes) using NLP applied to doctor-patient dialogue | Empirical evaluation with proposed pipeline, multi-stage NLP system | Multi-stage pipeline: ASR → speaker segmentation → utterance classification → note generation (T5/BART). | Dataset of 10,377 audio-recorded medical conversations annotated with SOAP notes | Technical |
| Thematic coding of interviews | Workflow implementation | To explore physicians' experiences with DAX Copilot (DAXC) | Qualitative study, semi structured interviews | Interviews analyzed via independent coding and thematic analysis. | Interview transcripts | Clinical |
| BLEU, METEOR, BERTScore, BLEURT | Model prototyping & Model development | Assess ChatGPT efficacy in drafting medical records and treatment plans in Japanese settings. | One of case study | Consultation transcript input into ChatGPT for SOAP notes; compared to physician-written summary (no metrics). | Single case | Technical |
| Perception of documentation burden, after-hours work, burnout risk and job satisfaction | Workflow implementation | Evaluate impact of Abridge (ambient AI) on clinicians’ documentation burden, burnout, after-hours work, and job satisfaction. | Pre- and post-implementation survey on administrative burden and burnout | Survey of physicians and advanced practitioners at KUMC assessing workflow, burnout, job satisfaction, and note completion before and after Abridge. | Surveys | Clinical |
| ROUGE-1, ROUGE-2, ROUGE-L, LINK recall, CORRECT recall, CORRECT accuracy | Model validation | To evaluate feasibility, performance, and accuracy of a digital scribe system for ED consultations | Experimental with model benchmarking and qualitative tagging | Tested T5, PEGASUS, BART models (zero-shot + fine-tuned) on 100 ED transcripts; evaluated with ROUGE metrics and annotation. | 100 ED phone consultations from Nationwide Children’s Hospital | Technical |
| ROUGE, UniEval, expert evaluation | Model validation | To assess the performance of LLMs' summarizing clinical dialogues using computational metrics and human evaluations | Experimental | Tested 5 summarization models (2 fine-tuned on clinical, 1 on general dialogues, 1 on long-book data, 1 general LLM). | 27 anonymized clinical dialogues | Technical |
| BLEU, ROUGE,METEOR scores, and BERTScore, Flesch-Kincaid grade Level - | Model prototyping & Model development | Identify opportunities for LLM zero-shot summarization in palliative care teleconsultations. | Controlled experiment | Quality evaluated with BLEU, ROUGE-L, METEOR, BERTScore; readability with Flesch-Kincaid grade. | bespoke doctor–patient conversation | Technical |
| BLEU, CHRF, METEOR, BERTScore, BLEURT, Levenshtein Edit Distance, WER, MER, WIL, Stanza, Snomed, ROUGE-1, ROUGE-2, ROUGE-3. ROUGE-4, ROUGE-L, ROUGE-WE, SkipThoughts, Embedding Avg, VectorExtrema, GreedyMatching, WMD, MoverScore | Model prototyping & Model development | To assess how well automatic metrics align with human evaluation in dialogues | Comparative experimental evaluation | Human rating of dialogue responses and correlation with automatic metrics. | DailyDialog, PersonaChat | Technical |
| Word count %, word count % (without stopwords), medical term %, speaker turn %, POS tagging. | Proposal of model/solution | Estimate how much GP-patient conversation is needed for a clinical summary to aid digital scribe design. | Clinician annotation of 44 transcripts | Manual coding and analysis of proportions of highlighted words, terms, and turns. | Audio-recorded and transcribed real GP consultations in Sydney | Technical |
| Physician Documentation Quality Instrument (PDQI-9). We compared the differences between the 3 methods in descriptive statistics, quantitative text metrics (word count and lexical diversity), the PDQI-9, Recall-Oriented Understudy for Gisting Evaluation scores - ROUGE1 & ROUGEL, and BERTScore. | Model prototyping & Model development | This study investigates the impact of a commercially available Dutch digital scribe system on clinical documentation efficiency and quality. | Mock set-up (consultations by actors, summary & evaluation by students) - manual, automatic and edited summaries | 22 students produced 430 summaries; quality compared across 3 methods using PDQI-9, word/lexical metrics, ROUGE, BERTScore. | Own set of mock-up consultations | Technical |
| Usability, clinician wellness, patient experience | Workflow implementation | To report the impact of a pilot implementation of this tool on clinicians’ documentation experience in the EHR and on overall well-being at Emory Healthcare | Pre- & postonboarding survey | Online surveys at onboarding and 60 days post-intervention. | Clinician self-reported survey data from Emory Healthcare participants | Clinical |
| SAIL, NASA Task Load Index | Model prototyping & Model development | Assess clinical utility of an ambient AI tool for consultation experience and documentation. | Part 2 of a multiphase study, full study is testing in... 1: sandbox environment, phase 2: simulated environment, 3: one site, multiple clinics, 4: multisite pilot in varying settings, 5: site wide rollout | Simulated consultations with actors and clinicians comparing AI vs EHR; documentation assessed by SAIL, clinician experience via questionnaires and NASA Task Load Index. | Audio recordings, transcriptions | Technical |
| Accuracy, fluency, completeness | Real-time model testing | To investigate the value of a note generation system and design an interface that best supports clinicians | Three phases of user research (Current Note-Taking Discovery, Initial UI Testing, Mock Consultations) and a live test | Interviews, surveys, Wizard-of-Oz prototype, mock-ups, live testing, analysis of generated notes. | Real-world consultations | Both |
| error characteristics: omissions, incorrect information, and additions. PDQI: (1) up to date, (2) accurate,  (3) thorough, (4) useful, (5) organized, (6) comprehensible, (7)  succinct, (8) synthesized, and (9) consistent | Model prototyping & Model development | Assess accuracy of ChatGPT-4 in generating SOAP notes vs gold standard transcripts. | Comparative evaluation of generated notes | Simulated encounters transcribed; ChatGPT-4 generated SOAP notes compared with chart review; errors classified; assessed with PDQI scores. | 14 simulated & handmade transcriptions | Technical |
|  |  |  |  |  |  |  |

| **Sample size** | **M/F** | **Population** | **Age** | **Other** | **ASR - Model used** | **ASR - performance** |
| --- | --- | --- | --- | --- | --- | --- |
| 10 | n/a | 5 hospitalists, 5 radiologists | n/a |  | n/a | n/a |
| 6 | 4m 2f | Physicians (evaluators), patient records from MIMIC-III | n/a |  | n/a | n/a |
| Train: 8,315, Dev: 1,039, Test: 1,023 conversations | n/a | Patients and physicians | n/a |  | Used but not specified in detail (transcriptions assumed to be provided) | n/a |
| 12 physicians | 8m, 4f | Clinicians | n/a |  | DAXC | n/a |
| 1 | 1f | Japanese | 66 | Medical history of colorectal cancer, diabetes, and obesity. She had been previously identified with an asymptomatic exacerbation of hyperthyroidism following the first dose of the BNT162b2 mRNA COVID-19 vaccine | Free application, transcript translated into English using Deepl | - |
| 181; Of the initial 181 clinicians who were offered access to the ambient AI documentation platform, 93 completed the pre-implementation survey (51.9% response rate). The post-implementation survey was completed by 99 of the 133 clinicians who were offered the survey (74.4% response rate). | 48m 51f | Clinicians from 30 medical specialties were included in the study. For analysis, these specialties were grouped into 3 categories: primary care, medical subspecialty, and surgical subspecialty. | 41 |  | Abridge | n/a |
| 100 transcripts | n/a | ED consultations from physicians and nurses | n/a | Each call consists of a multi-turn conversation (ranging  from 1 to 9 minutes conversation each) among PCTC nurses, an ED clinician or staff, and an external clinician or nurse | Amazon Web Services (AWS) Transcribe | Not directly quantified |
| 27 anonymized clinical dialogues | n/a | Clinicians | n/a |  | BART-LSG-conv | Highest ROUGE scores |
| 1 conversation | n/a | n/a | n/a | Carried out by research team using various models | n/a | n/a |
| 1000 dialogue turns evaluated by multiple annotators | n/a | Human annotators (non-specific demographics) | n/a |  | n/a | n/a |
| 44 consultations; 49 transcripts total | 15m 29f | Adult patients in primary care, mostly English-speaking, recruited via convenience sampling. | n/a | 28 were returning patients (63.6%), 16 were new (36.4%) | n/a | n/a |
| 21 | n/a | medical students with experience in clinical practice and clinical documentation from Leiden University Medical Center | n/a |  | Autoscriber - no specs mentioned | n/a |
| 117 onboarding, 55 follow-up; 31 both | 58.1% m | 32.3% being a primary care clinician | 48.4% between the ages of 20 and 39 | 64.5% as white, mean tenure as 8.4 years - see table for extension | Abridge | n/a |
| 8 clinicians, 5 medical consultants, 3 allied health professionals, 48 dummy patient stories | n/a | Clinicians | n/a |  | not specified | not specified |
| 7 clinicians in Phase 1, 5 in Phase 2, 5 in Phase 3, and 5 in the live test | n/a | UK clinicians with at least one year's experience at Babylon | n/a | In total, we collected 156 manual summaries, 137 automatic summaries, and 137 edited summaries from 21 students | Autoscriber - no specs mentioned | - |
| 14 transcripts, done 3 times in chatgpt, so 42 | n/a |  | n/a |  | Transcribed by hand | n/a |
|  |  |  |  |  |  |  |
|  |  |  |  |  |  |  |

| **NLP - Model used** | **NLP - performance** | **Results** | **Conclusion** |
| --- | --- | --- | --- |
| (FLAN-T5 (ref. 31), FLAN-UL2 (ref. 32), Alpaca33, Med-Alpaca34, Vicuna35 and Llama-2 (ref. 36)) and two proprietary (GPT-3.5 (ref. 37) and GPT-4 (ref. 38)). | Adapted GPT-4 outperformed specialists: 68% vs. 58% (specialists); GPT-3.5 scored 52%. | Summaries from best adapted LLM were equivalent (45%) or superior (36%) compared to clinicians. Adapted GPT-4 was more accurate than clinicians, domain adaptation significantly boosts QA performance | Adapted LLMs have the potential to support or even outperform specialists on complex clinical questions, but implementation requires caution and validation. |
| LLaMA 2, Mistral (fine-tuned) | Outperformed baselines in ROUGE/BERTScore; preferred by physicians | AsclepiAI outputs were more relevant, coherent, and clinically useful vs. baseline | LLMs are promising for real clinical tasks, but must be designed with care and tested rigorously. |
| T5, BART (fine-tuned) | T5 performed better than BART, ROUGE-L between 0.30–0.40 depending on section | Multi-stage NLP pipeline can generate clinically useful notes, factual accuracy still a challenge | Digital scribe systems are feasible but need improvements before clinical deployment. |
| DAX Copilot GPT-4 (OpenAI) | Some errors - hallucinations, misgendering, transcription mistakes | Overall, clinicians experienced reduced burden and anxiety, improved engagement, and greater ease in retaining details. However, issues such as hallucinations, unsolicited diagnoses, and transcription errors raised reliability concerns. | AI-driven documentation shows promise but needs improvements to increase reliability and broader applicability. |
| ChatGPT - 4 | A high similarity was observed between the content produced by the physician and the content generated by ChatGPT. | Similar output between physician & chatgpt | potential, but with limitations, such as hallucinations and reduced efficacy in specialized domains, important technology is integrated and serves as a complement, not a replacement. |
| Abridge | n/a | Clinicians who used ambient documentation perceived it as highly beneficial, reporting improved ease of documentation workflows (OR=6.91, 95% CI: 3.90–12.56, p<0.05). The intervention enhanced workflow efficiency within a short timeframe, reflecting strong user acceptance. | An ambient AI documentation platform had tremendous impact on improving clinician experience within a short time frame. Future studies should utilize validated instruments for clinician efficiency and burnout and compare impact across AI platforms. |
| T5-small, T5-base, PEGASUS-PubMed, BART-Large-CNN | BART-Large-CNN (best): ROUGE-1 F1=0.49, ROUGE-2 F1=0.23, ROUGE-L F1=0.35; Recall=71.4%, Accuracy=67.7% | BART-Large-CNN outperformed others, performance impacted by transcript quality, promising for assisting clinical documentation | Digital scribe shows potential, not ready to replace human input, more refinement and evaluation needed. |
| ChatGPT | Highest UniEval scores | ChatGPT outperformed other models, performance comparable to human summaries | LLMs can automate clinical dialogue summarization but face privacy challenges. |
| GPT-3.5, GPT-4 and LLaMA | GPT-4 has notable capacity for capturing longer sequences and maintaining semantic accuracy. | LLaMA2-7B achieved the highest BLEU score, while GPT-4 outperformed others in ROUGE-L, METEOR, and BERTScore. Readability analyses showed GPT-4’s summaries were moderately complex, while GPT-3.5 was most complex and LLaMA 70B closest to the reference grade level. | Our findings indicate that all the models have similar performance for the palliative care consultation, with GPT-4 being slightly better at balancing understanding content and maintaining structural similarity to the source, which makes it a potentially better choice for creating patient-friendly medical summaries. Threats and limitations of such approaches are also embedded in our analysis. |
| GPT-2, BlenderBot, DialoGPT | Compared via automatic metrics and human ratings | BLEURT and BERTScore showed stronger correlation with human ratings, BLEU and METEOR less reliable | Caution advised when using automatic metrics alone, human evaluation remains critical. |
| spaCy for POS tagging, MetaMap Lite for medical term recognition | No performance metrics reported | On average, 9.1% of all words, 26.6% of medical terms, and 27.3% of speaker turns were relevant, POS most relevant | Only a small portion (~20%) of conversation is used in summaries, future systems should focus on identifying this key fraction for digital scribes. |
| Autoscriber GPT 3.5, GPT 4 | n/a | Manual summarization took 202 seconds compared to 186 seconds for editing automatic summaries. Unedited AI outputs had lower PDQI-9 scores, higher word counts, and reduced lexical diversity, though clinicians noted ease of use. | This study shows digital scribes can improve documentation by providing draft summaries for physicians to edit, reducing time without compromising quality. Their usefulness may vary among physicians but could enhance the reusability of records. They hold promise in streamlining clinical documentation processes. Future research should evaluate their impact and quality in real clinical practice. |
| Abridge | n/a | This generative AI-based ambient listening documentation solution was associated with a significant increase in participants’ perception of documentation usability and patient experience. It also reduced perceptions of negative impact on well-being. | Generative AI-based documentation improves perceived usability, well-being, and patient interaction. Promising for EHR integration. |
| GPT-4 | Function well in scenarios with multiple speakers | AI-produced documentation achieved higher SAIL scores and shortened consultations by 26.3% without reducing patient interaction time. Clinicians also reported a more positive experience and lower task burden. | The AI tool significantly improved documentation quality and operational efficiency in simulated consultations. Clinicians recognised its potential to improve note-taking processes, indicating promise for integration into healthcare practices. |
| BART sequence-to-sequence model | n/a | Real-time note generation was preferred. Clinicians used the system differently based on their note-taking styles. The tool improved focus on patients by reducing time spent on documentation. | User experience studies revealed essential requirements for the design of a note generation system. |
| ChatGPT-4 | n/a | ChatGPT-4 generated SOAP-style notes but produced an average of 23.6 errors per clinical case, primarily omissions (86%). Accuracy varied across replicates and sections, with transcript length inversely correlated with note accuracy. | This study found high variability in errors, accuracy, and note quality with ChatGPT-4, limiting reliability. Errors varied across sections and replicates, with accuracy declining as transcripts grew longer and more complex. The generated notes do not meet standards required for clinical use, warranting caution before adoption. Further research is needed to improve accuracy and reduce errors before it can be considered a safe alternative. |
|  |  |  |  |
|  |  |  |  |
